# Socioeconomic changes post Indonesia-Papua New Guinea cross-border development

**DOI:** 10.3389/fsoc.2026.1794970

**Published:** 2026-04-23

**Authors:** Simon Abdi Kari Frank, Usman Idris, Marlina Flassy

**Affiliations:** Department of Anthropology, Faculty of Social & Political Sciences, Cenderawasih University, Jayapura, Indonesia

**Keywords:** border development, border tourism, cross-border trade, differential inclusion, ethnography approach

## Abstract

This study aims to examine how the development of the National Border Post (PLBN) Skouw reshapes socioeconomic dynamics in the Papua–Papua New Guinea border region. Using a qualitative ethnographic approach based on in-depth interviews, participant observation, and document analysis, the research identifies three primary areas of transformation: the intensification of cross-border trade, the emergence of border tourism, and the establishment of cross-border cultural festivals. The findings show that the development of PLBN Skouw has significantly accelerated economic activity and transformed the border from an isolated frontier into a dynamic socioeconomic hub. However, this transformation is not uniformly experienced. The study demonstrates that while PLBN-driven development has expanded economic opportunities, it simultaneously produces uneven participation and reinforces existing socioeconomic disparities, particularly affecting Indigenous Papuan communities. Rather than generating inclusive growth, border development operates through a pattern of differential inclusion, where access to opportunities is formally open but substantively unequal. This research contributes to border studies by advancing a critical perspective that integrates the Cross-Border Governance System framework with the concept of differential inclusion to explain how infrastructure-led development simultaneously enables integration and reproduces inequality. The study argues that the success of border development should not be assessed solely through economic growth indicators, but must also consider the distribution of benefits and the extent to which marginalized groups are able to participate meaningfully in emerging border economies. This finding highlights the need for justice-oriented border policies that prioritize equity alongside economic expansion.

## 1 Introduction

Historically, these border regions were often perceived as remote, underdeveloped areas primarily managed through a security-centric approach (Soleman and Noer, 2017). However, the past decade has witnessed a paradigm shift in Indonesia's border management strategy. This shift has been characterized by a transition from viewing borders merely as security perimeters to recognizing them as potential catalysts for economic development and regional integration. Central to this new approach is the construction of Cross-Border Posts (*Pos Lintas Batas Negara, PLBN*) at strategic locations along Indonesia's international borders with Malaysia, Timor-Leste, and Papua New Guinea (Rares, 2024).

Contemporary border management literature emphasizes the dual nature of border regions as both gateways for beneficial cross-border activities and barriers against detrimental elements (Sohn, 2014). This dualism is particularly relevant in the context of developing nations like Indonesia, where border regions often grapple with complex challenges including illegal trade, smuggling, human trafficking, and natural resource theft (Ohoiwutun et al., 2023).

The development of PLBNs represents an attempt to strike a balance between these competing imperatives. By providing controlled access points and improved infrastructure, PLBNs aim to facilitate legitimate cross-border economic activities while simultaneously enhancing the state's capacity to monitor and regulate border movements (Ptak et al., 2022). Within Papua, Indonesia's easternmost province, the development of PLBNs has been concentrated in two key locations: Jayapura in the north and Merauke in the south. These sites were selected based on several strategic considerations, including their relatively developed urban infrastructure, diverse populations, and potential for stimulating cross-border economic growth (Apriani, 2023).

The Skouw–Wutung Cross-Border Post, situated on the Indonesian side of the Papua Indonesia-Papua New Guinea border, has emerged as a focal point of Indonesia's border development strategy in the region. Since its inauguration, the PLBN Skouw has attracted increasing numbers of visitors, drawn not only by its functional role as a border crossing point but also by its impressive architecture and symbolic representation of Indonesian national identity (Arinto and Pidjath, 2015).

Recent research has greatly enriched knowledge of border politics by pointing to hybridities of border regimes with debordering and rebordering processes being waged at once. For example, (Su 2024) focuses upon the security–economy nexus when it comes to tourism, while (Su and Li 2021) show bordering processes to swing back and forth between openness and closure along the China–Myanmar border frontier. Comparable to Coleman's work is an examination of bordering selectivities at the U.S.–Mexico border by (Coleman 2005), who highlights entanglements of state control with cross-border flows. For Europe, shifts between open and closed border regimes and border landscapes are explored by (Wieckowski and Timothy 2021), with further reference by (Wieckowski 2023) to tripoint and symbolic border posts politics. Geographies of power beneath Southeast Asian cross-border development are explored by (Sparke et al. 2004), offering further complementarity to Laine's (2016) multiscalar border production program as local, national, and transnational scales are brought to bear upon one another. Debordering and rebordering are further theorized as mutual constitutions by (Herzog and Sohn 2019). Lastly, with an early foundational reference to various fashions with which borders have come to be theorized, equally relevant to Directly Relevant Categories with respect to all things Papua Indonesia-Papua New Guinea. (van Houtum 2000) offers a foundational work upon border theorization diversity. As a group, and when combined with other literature to follow, this body of work provides an enhanced theoretical framework through which PLBN Skouw can locally and globally contextualize its future trajectory as a border region to realize its vision to become a thriving tourism and economic border region at once.

Based on this body of literature, this research makes an effort to close key gaps by exploring economic changes after the formation of the PLBN Skouw in Papua. By integrating these wider theoretical perspectives with an ethnography-driven approach, this study seeks to provide new insights into how border infrastructure shapes economic activity, social relations, and cross-border interactions in peripheral areas defined by cultural diversity and uneven history. In doing so, this research not only advances empirical understanding about Papua Indonesia–Papua New Guinea border regions but also enriches theoretical debate about border hybridity by engaging with and engaging with perspectives from the Global South.

Building on this gap, this study adopts an analytical positioning that integrates border studies with economic anthropology to examine how infrastructure-led development reshapes economic practices in peripheral regions. Rather than treating economic change as merely a matter of quantitative growth, this research conceptualizes border economies as socially embedded processes, where trade, mobility, and value formation are negotiated through everyday interactions and institutional arrangements. In this context, the ethnographic approach is not only used to capture cultural dynamics, but also to interpret economic transformations as lived and relational experiences, revealing how different actors access, participate in, and benefit from emerging border economies. By combining the Cross-Border Governance System (CBGS) framework with the concept of differential inclusion, this study provides a nuanced understanding of how infrastructure development simultaneously enables economic integration and produces uneven participation. This positioning allows the research to move beyond descriptive accounts of border development and instead offer a theoretically grounded explanation of how economic value is produced, distributed, and contested in the Skouw–Wutung border region. This approach is particularly important in the Papua context, where economic processes cannot be separated from socio-cultural structures, historical marginalization, and asymmetrical power relations.

## 2 Literature review

This research reviews literature on border area development and socioeconomic transformation, focusing on border infrastructure, economic change, cultural dynamics, and cross-border tourism. It encompasses both theoretical frameworks and empirical studies on the socioeconomic impacts of border post development.

### 2.1 Border infrastructure development and economic change

The field of border infrastructure development has witnessed a significant evolution over the past decade, with a notable expansion beyond the conventional security-centric approaches to encompass a more expansive socioeconomic perspective. The conceptualization of borders as fluid spaces of interaction and not as rigid security boundaries (as proposed by Brunet-Jailly, 2011; Herzog and Sohn, 2019) is a notable advancement in this regard. The conceptual emphasis of this development study is a shift from the traditional security system approach, toward the development of supporting infrastructure that will make a significant contribution to the socioeconomic context. In alignment with this approach, (Wong Villanueva et al. 2022) posit that border infrastructure has a substantial influence on regional development, contingent upon the interconnection between formal institutions, informal networks, and physical infrastructure that facilitates cross-border economic integration. This approach has been demonstrated to be especially fruitful in instances where the establishment of new border facilities serves to stimulate economic transformation.

To strengthen the analytical foundation of border development studies, it is important to situate infrastructure-led transformation within broader patterns of border typology, disparity, and development dynamics. (Taubenböck et al. 2023) provide a comprehensive global perspective by demonstrating that border regions are not homogeneous spaces, but vary significantly in terms of economic integration, political function, and development trajectories. Their typology highlights how some borders function as growth corridors with high levels of economic exchange, while others remain peripheral and marginalized, characterized by structural inequalities and limited connectivity. In this sense, border infrastructure such as PLBN can act as a catalyst for transforming peripheral borderlands into more integrated economic zones, but the outcomes are highly contingent upon existing disparities and governance capacities. Applying this framework to the Papua context reveals that the Skouw–Wutung border operates within a condition of uneven development, where improved infrastructure coexists with persistent socioeconomic inequalities, particularly affecting Indigenous Papuan communities. Therefore, border development should be understood not only as a process of economic expansion, but also as a reconfiguration of spatial and social disparities, where integration and exclusion occur simultaneously. This perspective allows the study to move beyond general claims of development success and critically assess who benefits from border transformation and who remains excluded.

Empirical studies from a variety of geographic contexts provide evidence to support the assertion that border infrastructure development has the potential to effect transformative change. The research conducted in the border regions of Southeast Asia and Africa demonstrates that the enhancement of border facilities can facilitate local economic growth through the expansion of trade flows and market access, while also transforming informal cross-border trade into more regulated economic activity (Grundy-Warr and Schofield, 2005).

Nevertheless, this does not suggest that this approach is without critique. As highlighted by (Iglesias Ortiz and Karimi 2025), one of the key issues is that the development of state border areas can actually lead to increased disparities. In other words, while the development of physical border infrastructure facilitates differential inclusion, border infrastructure projects can inadvertently reinforce existing socioeconomic hierarchies, particularly impacting indigenous and marginalized communities.

Furthermore, from a tourism perspective, the emergence of border tourism as an economic driver represents a relatively new focus in the field of border studies, particularly within the context of developing countries. The literature on border tourism in developed areas such as Europe demonstrates that border infrastructure may become a tourist attraction in itself (Prokkola, 2019). Meanwhile, in developing countries, border infrastructure frequently continues to confront rudimentary challenges, including restricted accessibility and connectivity. Nevertheless, this can in fact facilitate the emergence of distinctive opportunities in the field of tourism development. In this context, the isolated and authentic characteristics of border areas may constitute a distinctive attraction for tourists seeking a differentiated experience. This potential can be optimized by the development of infrastructure that not only facilitates border administrative functions but also supports sustainable tourism activities and involves local communities as the primary stakeholders in these initiatives.

However, a significant knowledge gap remains with respect to the manifestation of this phenomenon in developing regions, in particular with regard to the participation of local communities and the distribution of benefits derived therefrom (Timothy et al., 2016). In addition to these perspectives, the literature on border landscapes provides valuable insights into the interrelation between space, culture, and identity at international frontiers. Classic studies of the U.S.–Mexico border (Arreola and Curtis, 1992; Martinez, 1994) demonstrate how built infrastructure, markets, and symbolic markers constitute landscapes of power and meaning, shaping everyday practices as much as formal economic exchange. Applying these insights to Papua suggests that PLBN development should not be viewed solely as a matter of economic facilitation, but also as the production of new symbolic geographies that influence how communities perceive and inhabit the border space.

This limitation is particularly pertinent when examining cases such as Papua, where tourism development interfaces with intricate social and cultural dynamics. The intricacy of border tourism development in Papua mirrors the broader difficulties encountered when integrating infrastructure development with the local socio-cultural milieu. The distinctive character of the Papuan context, with its rich traditional culture and distinctive social structure, requires a more contextualized approach to the development of cross-border tourism. This necessitates a profound comprehension of the ways in which tourism infrastructure might be devised in order to foster the maximum possible participation of indigenous communities, while guaranteeing that the resultant economic gains are dispersed in a manner that is equitable. This gap in understanding becomes increasingly critical, particularly in light of the potential of tourism to serve as an instrument for the empowerment of local communities. However, failure to manage the associated socio-cultural sensitivities could also result in the emergence of new tensions.

### 2.2 Cultural dynamics and social integration in border areas

The challenge of balancing economic development aspirations with the preservation of cultural values in border regions reflects a broader dilemma inherent in the process of modernization. In particular, this illustrates the tension between the necessity of developing infrastructure and the importance of preserving the socio-cultural identity of border communities. The concept of “border hospitality” has recently emerged as a significant theoretical development in our understanding of cultural dynamics at the border. This concept offers valuable insights into how formal border structures can facilitate informal social dynamics and cultural exchange (Gubrium et al., 2025). Empirical research conducted in a variety of geographical and cultural contexts indicates that the relationship between border development and the dynamics of cultural processes is complex. This approach offers an alternative perspective that acknowledges the potential for border infrastructure to function not only as a barrier, but also as a facilitator of cross-border socio-cultural exchange.

Nevertheless, critical perspectives from scholars indicate the potential for tensions between economic development and cultural preservation in border regions (Laven and Baycroft, 2008). The necessity for a development approach that strikes a balance between economic opportunity and cultural sustainability is highlighted by this work. The implementation of this concept in a development context necessitates a profound comprehension of the local dynamics and the mechanisms through which communities adapt to change. This necessitates an exhaustive examination of the social interaction patterns that have been established, the dominant value systems, and the societal capacity to adapt to structural transformations. The principal challenge is to devise infrastructure and development programs that not only optimize economic potential, but also reinforce and safeguard existing socio-cultural networks in order to create a genuinely sustainable and contextualized model of development.

In the context of the border between Papua Indonesia and Papua New Guinea, the development process has led to the formalization of cross-border economic opportunities, including trade and the potential for tourism. On the other hand, transformation has the potential to intersect with long-standing traditional practices and cross-border kinship networks that have existed between the same ethnic groups in both countries over a significant period of time. The particular challenge in the region is to integrate modern infrastructure with the traditional mobility patterns and socioeconomic exchange practices of indigenous peoples, taking into account the existence of strong cross-border kinship ties, in particular among those tribes that have inhabited the region for centuries, long before the demarcation of modern state borders. An approach to development that fails to consider these socio-cultural factors may inadvertently give rise to new tensions, particularly in instances where regulatory frameworks conflict with deeply entrenched traditional practices.

This discussion resonates with the border landscape literature, which emphasizes that borders are cultural as well as territorial constructions. (Arreola and Curtis 1992) argue that border landscapes emerge through the interplay of material structures and cultural representation, while (Martinez 1994) highlights the everyday practices that sustain cross-border ties despite political demarcation. For the Papua Indonesia–Papua New Guinea context, such perspectives illuminate how PLBN Skouw embodies both an architectural assertion of sovereignty and a lived space where Indigenous kinship networks and cultural exchange persist. Recognizing this duality is crucial for designing development strategies that respect socio-cultural sustainability alongside economic growth.

## 3 Research method

### 3.1 Research design

This study adopts an ethnographic approach to examine socioeconomic transformations following the construction of the Skouw Border Post (PLBN). The choice of ethnography is directly linked to the study's primary objectives, namely to analyze how border infrastructure reshapes economic practices, social interactions, and community perceptions within the frameworks of the Cross-Border Governance System (Wong Villanueva et al., 2022), differential inclusion (Iglesias Ortiz and Karimi, 2025), and border conviviality (Gubrium et al., 2025). In other words, this methodological choice enables the research to capture both economic and cultural-symbolic dimensions of change, as emphasized in the literature review.

### 3.2 Data collection methods

This study employed ethnographic techniques to understand economic changes in the Skouw border region. Participant observation focused on: (1) cross-border trade, (2) border tourism, and (3) cross-border festivals. Observations included market activity, border mobility, and the symbolic functions of the PLBN in daily life. Semi-structured interviews were conducted with traders, community leaders, officials, and visitors from PNG. Questions were guided by concepts such as differential inclusion, symbolic sovereignty, and conviviality.

### 3.3 Sampling strategy

The study employs purposive and snowball sampling techniques to identify key informants and ensure a diverse range of perspectives are captured. Participants were selected from diverse social, economic, and cultural backgrounds within the region. The sampling emphasized inclusivity, particularly for marginalized voices, to capture a fuller picture of local transformation. A profile of informants is presented in Table 1.

**Table 1:** Research informants.

No.	Informant code	Gender	Age	Role
1	INF 01 CW	M	65	Chief of Nyao
2	INF 02 AW	M	45	Mosso village chief
3	INF 03 MSR	F	34	Skouw market traders
4	INF 04 AK	M	63	Chief of Skouw
5	INF 05 SR	F	48	Local government officials
6	INF 06 DB	M	40	Mosso village residents
7	INF 07 AT	M	41	Officials guarding the border
8	INF 08 HR	F	48	Buyers from Papua New Guinea
9	INF 09 LTB	M	53	Skouw Village Residents
10	INF10 YL	F	36	PBLN Skouw–Wutung visitors

Source: Research Team Data Processing, 2025.

The distribution of informants reflects a deliberate effort to capture diverse perspectives across key stakeholder groups in the border economy. Of the ten informants, three were directly involved in cross-border trade activities (traders and market participants), three represented local authority and governance structures (village chiefs and government officials), two were community members residing in border villages, and two were external actors engaging in cross-border mobility (including visitors and buyers from Papua New Guinea). This categorization was designed to ensure representation of both economic actors and institutional stakeholders, as well as cross-border participants who actively shape the dynamics of exchange at the PLBN.

The sample size was determined based on the principle of theoretical saturation, where data collection was continued until no substantially new information or themes emerged from additional interviews (Guest et al., 2006; Creswell and Poth, 2018). Given the focused scope of the study and the relatively bounded social setting of the Skouw border area, ten informants were considered sufficient to capture recurring patterns and key variations in economic and social experiences. This approach aligns with qualitative ethnographic research standards, which prioritize depth of insight and contextual richness over numerical generalization (Spradley, 1980; Hammersley and Atkinson, 2007). Furthermore, the use of purposive and snowball sampling ensured that participants were selected based on their relevance to the research objectives, particularly their involvement in trade, governance, and cross-border interactions (Patton, 2015).

### 3.4 Data analysis

Data analysis was conducted iteratively alongside data collection, enabling real-time refinement of research focus. The analytical process followed an ethnographic approach involving domain, taxonomic, and thematic analysis, enabling systematic interpretation of patterns within social and economic practices (Spradley, 1980). Thick descriptions were developed from observations and interviews, which were then systematically coded using thematic and constant comparative methods. These methods allowed identification of key patterns and construction of conceptual themes. Triangulation across field notes, interviews, and observations enhanced the reliability and depth of interpretation. This methodological approach ensures analytical rigor while maintaining sensitivity to the socio-cultural context of border communities.

### 3.5 Ethical considerations

This study followed strict ethical protocols appropriate for research in sensitive border areas. Informed consent was obtained from all participants, and confidentiality and anonymity were consistently upheld. The researcher respected local cultural norms and power dynamics throughout the fieldwork. Ethical integrity was further reinforced through member checking and feedback sessions, allowing participants to validate the representation of their experiences. Additionally, the researcher maintained continuous reflexive awareness of their positionality as an outsider, recognizing how their presence and interpretations could influence the research process and community dynamics.

### 3.6 Reflexivity

Reflexivity plays a critical role in this ethnographic study, recognizing the researcher's positionality and the subjective nature of qualitative inquiry. Throughout the research, a reflexive journal was maintained to record personal reactions, shifting interpretations, and evolving researcher-community dynamics. This practice enhanced research transparency, supported data interpretation, and served as a continuous tool for self-awareness amid the ethical and methodological complexities of conducting fieldwork in a sensitive border context.

## 4 Results and discussion

### 4.1 Intensification of cross-border trade

The development of PLBN Skouw has significantly enhanced cross-border trade activities, aligning with the Indonesian government's strategic objective of stimulating economic growth in border regions (Arinto and Pidjath, 2015; Timisela, 2015). This intensification of trade is manifested through several key developments. The relocation and substantial improvement of the Skouw market represent a cornerstone of this economic transformation. The integration of the market into the PLBN complex has created a more structured and economically efficient environment for commercial activities, addressing previous infrastructural inadequacies (Brunet-Jailly, 2011; Herzog and Sohn, 2019; Grundy-Warr and Schofield, 2005). This development aligns with Wong Villanueva et al.'s (2022) Cross-Border Governance System (CBGS) framework, demonstrating how targeted infrastructure development can facilitate more effective cross-border economic integration (also see Brunet-Jailly, 2011; Wong Villanueva et al., 2022). However, this intensification should be understood not merely as a linear increase in trade volume, but as part of a broader structural transformation of the border economy.

**Figure 1:**
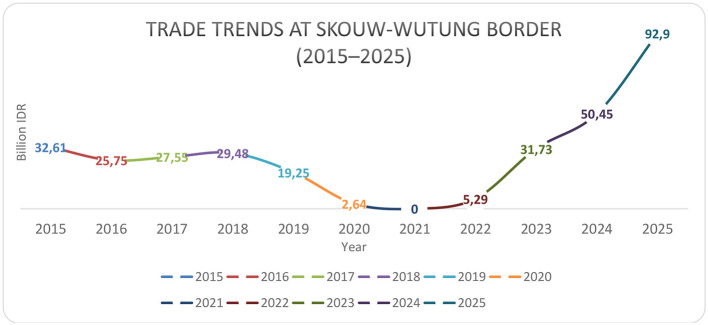
Trade trends at Skouw–Wutung border (2015–2025). Source: researcher data processing sources from various sources^1^.

Furthermore, the expansion of official market days from two to three per week (now including Tuesday, Thursday, and Saturday) is indicative of growing economic activity and demand (Marlissa et al., 2021; Elabi, 2018). This increase in trading frequency not only provides more opportunities for economic exchange but also strengthens the socioeconomic ties between communities on both sides of the border, contributing to what (Gubrium et al. 2025) term “border conviviality.” Yet, beyond the descriptive growth in trade days, this pattern reflects a deeper restructuring of border governance: infrastructures designed to stimulate trade simultaneously shape who has access to opportunities and who faces structural constraints (Gubrium et al., 2025; Herzog and Sohn, 2019). Thus, the PLBN acts as both a facilitator of integration and a regulator of participation, underscoring the dual nature of state-led border development. To complement these observations, Figure 1 presents the trend of cross-border trade at the Skouw–Wutung border from 2015 to 2025.

Trade trends at the Skouw–Wutung border crossing for the period 2015–2025 show a pattern of moderate fluctuations before the pandemic, followed by extreme contraction, and then a very rapid recovery. In the initial phase (2015–2019), trade values were relatively stable with a gradual upward trend from IDR 32.61 billion (2015) to around IDR 29.48 billion (2018), before declining slightly to IDR 19.25 billion in 2019. This pattern reflects the evolving dynamics of cross-border trade shaped by infrastructural, regulatory, and institutional factors. Nevertheless, this period can generally be categorized as an economic baseline phase, during which trade activity is operating normally and indicates the potential for relatively stable growth before any major external disruptions.

Regarding the construction of the Skouw PLBN, which began in 2016 and was inaugurated in 2017, the pre- and post-construction phases show quite distinct differences. Prior to construction (2015–2016), trade remained stagnant and showed no significant acceleration, indicating limited infrastructure and a suboptimal border management system. After the PLBN became operational

(2017–2019), trade stability and capacity began to improve, reflected in the relatively high and maintained value of nearly IDR 30 billion. This demonstrates that the PLBN's presence plays a role in improving border governance, increasing the flow of goods, and strengthening cross-border economic connectivity. Although its impact has not been fully maximized due to external factors such as cross-border regulations and local economic structures.

The most significant disruption occurred in 2020–2021, when trade value dropped drastically to near zero in 2021 due to border closures during the COVID-19 pandemic. However, this phase proved a turning point, confirming the economic resilience of border regions. Following the reopening in 2022 (IDR 5.29 billion), there was a significant surge in 2023 (IDR 31.73 billion) and 2024 (IDR 50.45 billion), reaching IDR 92.9 billion in 2025. Further analysis reveals that this post-pandemic acceleration reflects not only a rebound but also the full optimization of the Skouw PLBN's function as a strategic economic infrastructure that had previously been underutilized. Therefore, a comparison of the pre- and post-PLBN construction indicates that the infrastructure's presence is an enabling factor that only truly demonstrates a significant impact when supported by normal mobility conditions. Overall, this pattern illustrates the transformation from a shock economy to a high-growth border economy, while also confirming that border infrastructure investment has a long-term, accelerating effect on cross-border trade growth.

From a border typology perspective, this trajectory reflects a transition from a peripheral and semi-formal trading zone toward an emerging cross-border growth corridor. As highlighted by (Taubenböck et al. 2023), such transformations are shaped not only by infrastructure expansion but also by pre-existing disparities and governance capacities. In the Skouw case, while the PLBN has significantly enhanced connectivity and market accessibility, the benefits of this transformation remain unevenly distributed. This indicates that economic integration operates alongside differential inclusion, where certain actors—particularly mobile and well-capitalized traders—are better positioned to capture emerging opportunities than Indigenous Papuan communities. Consequently, the observed intensification of trade should be interpreted as a dual process of growth and stratification, rather than a uniformly inclusive development outcome. These quantitative trends provide a macro-level overview of trade dynamics, which is further explored through ethnographic findings; in this regard, INF 02 CW revealed the following:

“*Before there was the National Border Post Skouw-Wutung, our village was rather quiet, now every week many people come.”*

Then the next informant INF 08 HR who is a resident of Papua New Guinea said as follows:

“*We came to Skouw market every Tuesday, Thursday and Saturday because the goods here were cheap and good. I usually bought our daily needs.”*

Then the informant INF 03 MSR as a trader at Skouw market said:

“*Since this market opened near the post, our goods have been selling better. Many PNG people came, local residents also shop more frequently due to improved accessibility.”*

These testimonies highlight how local actors perceive the benefits of PLBN-related developments. This suggests that perceptions of economic improvement are embedded within asymmetrical structures of access, where visibility of growth may mask underlying inequalities. Yet, rather than treating these quotes as self-evident proof of success, they should be read within the broader state-promoted discourse of border modernization. While informants stress increased activity and accessibility, such narratives may obscure the uneven distribution of benefits, especially between established traders with resources and small-scale Indigenous sellers who remain vulnerable to competition. This requires a more critical reading of informant voices as situated within structures of opportunity and exclusion.

The market's diverse product range, encompassing everything from basic necessities to electronic goods, caters to a broad spectrum of consumer needs on both sides of the border. This diversity in trade goods underscores the market's role in fulfilling various economic functions, from meeting basic needs to facilitating access to more sophisticated consumer products. The acceptance of both Indonesian Rupiah and Papua New Guinean Kina in trade transactions further lubricates these economic exchanges, reducing currency exchange barriers and fostering a more integrated cross-border economic space. However, the observed asymmetry in trader demographics, with a notable disparity between immigrant traders (primarily of Bugis-Makassar and Javanese origins) selling imported goods and Indigenous Papuan traders focusing on local commodities, raises important questions about economic equity and inclusion. This imbalance resonates with Iglesias Ortiz and Karimi's (2025) concept of “differential inclusion,” where border regimes formally extend access to all, but in practice create hierarchies of participation (Iglesias Ortiz and Karimi, 2025; Brunet-Jailly, 2011). The Skouw case demonstrates that while the PLBN enables convivial interactions and increases market vibrancy, it simultaneously reproduces socioeconomic stratifications, privileging mobile, well-capitalized migrant traders over less resourced Papuan vendors (Grundy-Warr and Schofield, 2005; Herzog and Sohn, 2019). This paradox reveals how border development can succeed economically while deepening inequalities unless targeted interventions are introduced to empower marginalized groups. This reinforces the argument that border infrastructure does not inherently produce inclusive development, but rather reconfigures existing inequalities within a new economic landscape.

### 4.2 Emergence of border tourism

The unforeseen emergence of PLBN Skouw as a tourist destination represents a significant, if unintended, outcome of the border post's construction. This phenomenon aligns with global trends in border tourism, where unique border features and experiences attract visitors seeking novel cultural and geographical encounters (Livandovschi, 2017; Mayer et al., 2019; Prokkola, 2019; Su, 2024). The architectural grandeur of the PLBN building, with its synthesis of national symbols and local Papuan motifs, serves as the primary draw for this emergent tourism. This architectural approach not only creates an aesthetically appealing landmark but also symbolically reinforces the nation's presence at its periphery, a strategy often employed in border regions to assert sovereignty through visual means (Sofield, 2006; Martinez, 1994). Beyond aesthetics, this architectural symbolism can be interpreted as a performative act of the state, transforming the border from a marginal space into a stage for nationhood. The attraction for visitors lies not only in the building's beauty but also in the way it materializes state authority, turning border infrastructure itself into a consumable tourist spectacle (Arreola and Curtis, 1992).

**Figure 2:**
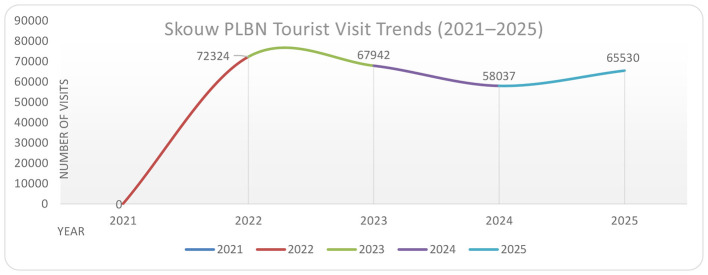
Tourism visits to Skouw border post (PLBN Skouw), 2021–2025. Sources: the National Border Management Agency of the Republic of Indonesia (BNPP RI) 2026^2^.

The significant improvement in accessibility, particularly through the completion of the Hamadi-Holtekamp Bridge, has been instrumental in facilitating this tourist influx. This infrastructural development has effectively shrunk the psychological distance between the border region and Jayapura City, making what was once perceived as a remote frontier more readily accessible to domestic and international visitors alike (Brunet-Jailly, 2011; Wong Villanueva et al., 2022). To illustrate this increase in tourist mobility, Figure 2 presents the trend of visitor numbers at PLBN Skouw from 2021 to 2025.

The trend of tourist visits to the Skouw Border Crossing (PLBN) exhibits a non-linear dynamic, but rather shifts through phases of disruption, acceleration, and structural adjustment. 2021 can be understood as a period of “collapse mobility,” where border closures due to the COVID-19 pandemic brought visitation to a virtual standstill. This phase should not be understood merely as a decline, but reflected the high dependence of border tourism on cross-border mobility regimes. When restrictions were relaxed in 2022, there was a significant surge in visits, reaching 72,324 people, indicating both accumulated pent-up demand and post-reopening euphoria. This surge not only reflects recovery but also indicates that the Skouw Border Crossing (PLBN) has repositioned itself as an attractive tourist destination, rather than simply a border control point. However, this phase was not stable, as a gradual decline in visitation in 2023 and 2024 was observed, indicating a process of normalization after the initial euphoria subsided and visitation patterns began to return to a more realistic and sustainable rhythm.

Nevertheless, the renewed increase in the number of visits in 2025, reaching 65,530, indicates that the Skouw PLBN area is entering a consolidation phase as a relatively stable border tourism destination. This increase is no longer driven by the shock effects of the reopening phase, but rather by the strengthening of structural factors such as improved accessibility, tourism promotion, and the growing recognition of the PLBN as a new tourism icon in the border region. Thus, the resulting visitation patterns not only reflect mobility dynamics but also demonstrate the complex interactions between cross-border policies, tourist perceptions, and regional economic integration. In this context, the Skouw PLBN is transforming from a mere administrative infrastructure into a hybrid space combining control functions, a symbol of sovereignty, and a tourist destination. Therefore, the sustainability of this positive trend will depend heavily on the management's ability to maintain a balance between security, open access, and economic inclusiveness for local communities, particularly Indigenous Papuans. The sharp increase after 2021 reflects the rapid normalization of mobility and the growing attractiveness of PLBN Skouw as a border tourism destination.

From the perspective of border development dynamics, the evolution of tourism at PLBN Skouw reflects a functional shift from a controlled border zone to a hybrid socioeconomic space. However, consistent with broader border typologies, this transformation is not uniformly experienced across all social groups. While increased visitor numbers indicate growing integration into regional tourism circuits, the limited involvement of Indigenous Papuans highlights persistent structural disparities. This suggests that tourism development, similar to trade expansion, operates within a framework of differential inclusion, where visibility and accessibility do not necessarily translate into equitable participation. These macro-level patterns are further reflected in the lived experiences of local stakeholders. One of the research informants INF 05 SR who is an employee at the local government, said as follows:

“*Now if there are guests from outside, for example official guests from outside come to Jayapura. one of the landmarks visited is PLBN Skouw-Wutung. because the place was highly Instagrammable so suitable for posting on social media.”*

Then the informant INF 07 AT as an Indonesian National Army officer guarding the Border added as follows

“*Since the PLBN was built, many visitors have come primarily to take photographs. but we also checked the purpose of the visit. if the situation was conducive, we allowed it.”*

These narratives illustrate how tourism in Skouw is increasingly shaped by digital culture, particularly the pursuit of “instagrammable” experiences (Timothy et al., 2016). However, this trend also reveals a shift in the meaning of borders: from militarized zones of control into commodified spaces of leisure. The irony is that security officers remain gatekeepers of access, underscoring that tourism and surveillance coexist uneasily in the same physical space.

The opportunity for visitors to briefly cross into Papua New Guinea offers a unique international experience within a confined geographical space. This aspect of border tourism capitalizes on what (Jiang and van der Velde 2025) describe as the “geographical imagination” of visitors, where the act of border crossing, however brief, fulfills a desire for transnational mobility and experience. The proliferation of “Instagrammable” spots within the PLBN complex reflects a savvy, if perhaps unintended, adaptation to contemporary tourism trends driven by social media (Su and Li, 2021). This aspect of the site's popularity, particularly among younger visitors, underscores the evolving nature of tourist experiences and the growing importance of digital media in shaping travel destinations and behaviors.

However, the apparent limited participation of local communities, particularly Indigenous Papuans, in tourism-related economic activities is a concern that echoes the asymmetries observed in cross-border trade. This disparity suggests a need for more inclusive development strategies that ensure local communities can effectively capitalize on the economic opportunities presented by this emerging tourism sector (Iglesias Ortiz and Karimi, 2025). This exclusionary pattern demonstrates that while PLBN tourism succeeds in attracting visitors, it risks reproducing socioeconomic disparities by marginalizing local actors. The commodification of border space without parallel inclusion of Indigenous stakeholders transforms them into passive onlookers of a development narrative enacted on their land (Gubrium et al., 2025). Unless policy interventions address this imbalance, border tourism could reinforce a form of symbolic dispossession, where the border becomes both a spectacle for outsiders and a missed opportunity for those who live closest to it. In this sense, border tourism at Skouw represents not only an economic opportunity, but also a contested space where inclusion and exclusion are continuously negotiated.

### 4.3 Establishment of cross-border festivals

The annual Cross-Border Skouw–Wutung Festival represents an innovative strategy to strengthen cultural and economic exchange in the border area. As a cultural platform, it plays a key role in preserving and promoting local traditions through performances and handicrafts, which helps maintain cultural identity amid globalization and social transformation (Putri et al., 2024; Laven and Baycroft, 2008; Arreola and Curtis, 1992). Economically, it provides opportunities for small and medium enterprises to showcase and sell their products, contributing to local livelihoods. Yet, the festival is not only a space for celebration, but also a state-mediated performance of multiculturalism designed to project harmony across the border (Brunet-Jailly, 2011; van Houtum, 2000). In this dual function, the event both empowers local communities and simultaneously operates as a tool of soft power to support border diplomacy. The festival's role in fostering cross-border cooperation and understanding between communities on both sides of the border is perhaps its most significant contribution. This aspect of the event exemplifies Gubrium et al.'s (2025) concept of “border conviviality,” creating a space for positive cross-border interactions and relationship-building that transcends the typically rigid structures of border control and national sovereignty (Gubrium et al., 2025; Prokkola, 2019). As a tourism attraction, the festival has become a draw for domestic and international visitors, further cementing the area's emergence as a tourist destination. This development aligns with research highlighting the role of cultural events in promoting tourism and local economic development (Satria and Erlando, 2018; Timothy et al., 2016; Livandovschi, 2017). However, this conviviality must be critically examined: while festivals temporarily suspend the rigidity of the border, they may simultaneously mask persistent inequalities. Participation tends to be more accessible to established artisans and traders, leaving younger or less connected Papuan entrepreneurs at the margins of these opportunities. This indicates that conviviality, while genuine in moments of encounter, is uneven in its distribution of benefits (Iglesias Ortiz and Karimi, 2025; Herzog and Sohn, 2019). Additionally, this events demonstrates how cultural capital can be leveraged for economic gain in border regions. The research informant INF 01 CW as the head of the Nyao tribe living on the border said as follows:

“*This festival was important to show our culture. We can be connected with our families in PNG. But young people should be more involved.”*

Then, added by the head of the Skouw Tribe INF 04 AK as follows:

“*The festival made selling more crowded. Many people came to buy noken and food. But we need training so that our business can progress.”*

These voices highlight both the pride and the challenges felt by community leaders. On one hand, festivals reaffirm cultural belonging and kinship ties across the border; on the other, they expose structural gaps in capacity building, particularly for youth and small traders. The desire for training indicates that cultural capital alone is insufficient without supportive policies that enhance skills, market access, and sustainability (Wong Villanueva et al., 2022). This resonates with debates on “cultural commodification,” where heritage is celebrated but not necessarily converted into equitable livelihoods for Indigenous actors (Sohn, 2014; Sparke et al., 2004).

The construction of PLBN Skouw has catalyzed multifaceted and significant economic and social changes in the Papua-Papua New Guinea border region. These changes have effectively transformed the area from an isolated frontier to a dynamic hub of cross-border activity, tourism, and cultural exchange. However, the findings also highlight potential areas for improvement, particularly in ensuring equitable participation of Indigenous Papuans in the emerging economic opportunities. The observed transformations align with various theoretical frameworks in border studies, from the CBGS framework's emphasis on cross-border governance and integration to concepts of differential inclusion, geographical imagination, and border conviviality. However, they also reveal the complex and sometimes contradictory outcomes of border development initiatives, where increased economic activity and cross-border integration can coexist with persistent or even exacerbated social and economic inequalities.

This pattern further illustrates that border development in Skouw is not limited to economic transformation, but also involves the reconfiguration of cultural and symbolic spaces. In line with border typology frameworks, cultural events such as festivals function as mechanisms of soft integration, yet remain embedded within unequal structures of access and participation.

Taken together, the findings across trade, tourism, and cultural exchange demonstrate that the development of PLBN Skouw has fundamentally transformed the border from a peripheral zone into a dynamic node of economic and social interaction. However, this transformation is not uniform, but characterized by uneven participation and differentiated access to opportunities. This confirms that border development operates through a dual logic of integration and inequality, where infrastructure expansion simultaneously enables growth and reproduces structural disparities. Therefore, the Skouw case contributes to broader debates in border studies by showing that the success of border development cannot be assessed solely through economic indicators, but must also consider the distributional consequences across different social groups.

## 5 Conclusion

This study identified three significant transformations following the development of PLBN Skouw: intensified cross-border trade, emerging border tourism, and the rise of cross-border cultural festivals. These changes reflect a shift in the border's role from isolated frontier to dynamic socioeconomic hub. However, unequal participation, especially among Indigenous Papuans, raises critical concerns regarding inclusivity and distributive equity. These findings can be further understood through the lens of social justice and inclusion theory, which emphasize not only access to economic opportunities but also the fair distribution of benefits and meaningful participation in development processes. In the context of Skouw, the expansion of cross-border trade and tourism has increased overall economic activity; however, this growth has not been experienced equally across all groups. Indigenous Papuans, in particular, remain positioned within structurally constrained roles, reflecting patterns of unequal access and limited capacity to benefit from emerging opportunities. This suggests that border development, while successful in generating economic dynamism, simultaneously reproduces existing inequalities unless accompanied by targeted inclusive policies.

The research findings support and enrich various theoretical frameworks. Importantly, these findings also extend the discussion on differential inclusion by demonstrating that access to border economies is not uniformly distributed, but structured by social position, mobility, and economic capital. This reinforces the argument that inclusion must be understood not only as formal participation, but as substantive and equitable engagement within economic systems. In this regard, the Skouw case highlights the need to move beyond growth-oriented metrics toward justice-oriented development frameworks. Including cross-border governance systems, differential inclusion, and border conviviality—highlighting how borders serve as both connectors and dividers in everyday life. Moving forward, development strategies should emphasize local empowerment, equitable access, and sustainable cultural integration to ensure that border transformation benefits all communities equally. PLBN Skouw illustrates that infrastructure, when socially attuned, can reshape borderlands into spaces of cooperation and opportunity.

Therefore, the transformation of the Skouw border should be interpreted not only as a success of infrastructure-led development, but also as a critical site where questions of social justice, inclusion, and inequality are negotiated. This underscores the importance of integrating economic development strategies with socially responsive policies that prioritize local empowerment, capacity building, and equitable access for Indigenous communities. Without such interventions, border development risks becoming a mechanism that amplifies, rather than reduces, existing socioeconomic disparities.

While this study provides valuable insights into the economic changes following the construction of PLBN Skouw, it is important to acknowledge its limitations. The ethnographic approach, while offering rich contextual data, may limit the generalizability of findings to other border contexts. Moreover, the study captures only short-term changes; long-term impacts remain uncertain. Future research should explore the causes of economic participation gaps, their solutions, and the environmental effects of increasing cross-border activity.

## Data Availability

The raw data supporting the conclusions of this article will be made available by the authors, without undue reservation.

## Footnotes

1 Dinas Perindustrian dan Perdagangan Provinsi Papua (Papua Province Industry and Trade Office, Indonesia), (2017); Konsulat Republik Indonesia di Vanimo (Performance Report of the Indonesian Consulate in Vanimo), (2022); Marlissa et al. (2021); Manibor (2022); Thane et al. (2023); Badan Nasional Pengelola Perbatasan Republik Indonesia (National Border Management Agency of the Republic of Indonesia), (2025); Evarukdijati (2026).

2 Available at: https://bnpp.go.id/ accessed on March 27, 2026. Data used as needed and adjusted for website searches.
